# Acute Paralytic Ileus Induced by Quetiapine: A case Report

**DOI:** 10.1192/j.eurpsy.2023.1176

**Published:** 2023-07-19

**Authors:** M. F. Tabara, K. N. Aykaç

**Affiliations:** 1 Psychiatry; 2 İnternal Medicine, Bingol State Hospital, bingol, Türkiye

## Abstract

**Introduction:**

Paralytic ileus is the slowing or complete cessation of the passage of intestinal contents without a barrier to prevent passage in the gastrointestinal tract. Many factors such as heavy metal poisoning, infections, metabolic instabilities, spinal cord injuries, drugs and post-operative reasons can cause paralytic ileus. Quetiapine is a second generation antipsychotic drug acting on multiple receptors. Due to its muscarinic receptor antagonism, adverse effects on the gastrointestinal tract may occur.

**Objectives:**

In this case report, acute paralytic ileus developing in a patient with bipolar disorder who was being treated with sodium valproate and quetiapine is discussed.

**Methods: Case:** The case is a 60-year-old male patient diagnosed with bipolar disorder. Apart from the medical diagnoses of hypertension and coronary artery disease, he had no other additional illness or history of surgery. He was brought to the emergency department in March 2022 with complaints of nausea, vomiting, abdominal distension, and decreased oral intake.

**Results:**

There are few case reports of paralytic ileus associated with quetiapine in the literature. In a study published in 2018, it was reported that paralytic ileus developed on the 15th day following the initiation of quetiapine therapy (Chiang & Lan, Clin Psychopharmacol Neurosci 2018; 16(2) 228–231). In another case report published in 2016, it was shown that ischemic colitis developed in a patient using quetiapine and tropatepine drugs (Cuny et al., *L’encephale 2016; 43*(1) 81–84). In the case we reported, the patient had been using quetiapine for about 5 years. Long illness duration and old age were risk factors for the emergence of paralytic ileus in our case.

**Image:**

**Figure:**
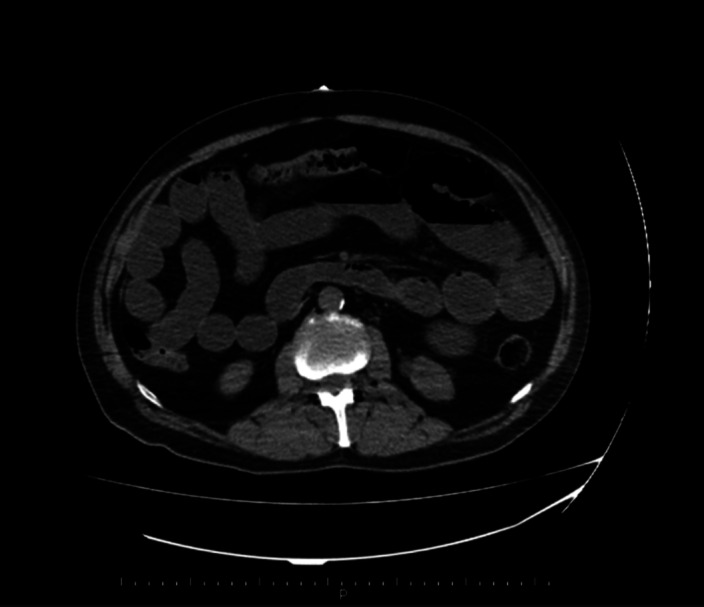

**Image 2:**

**Figure:**
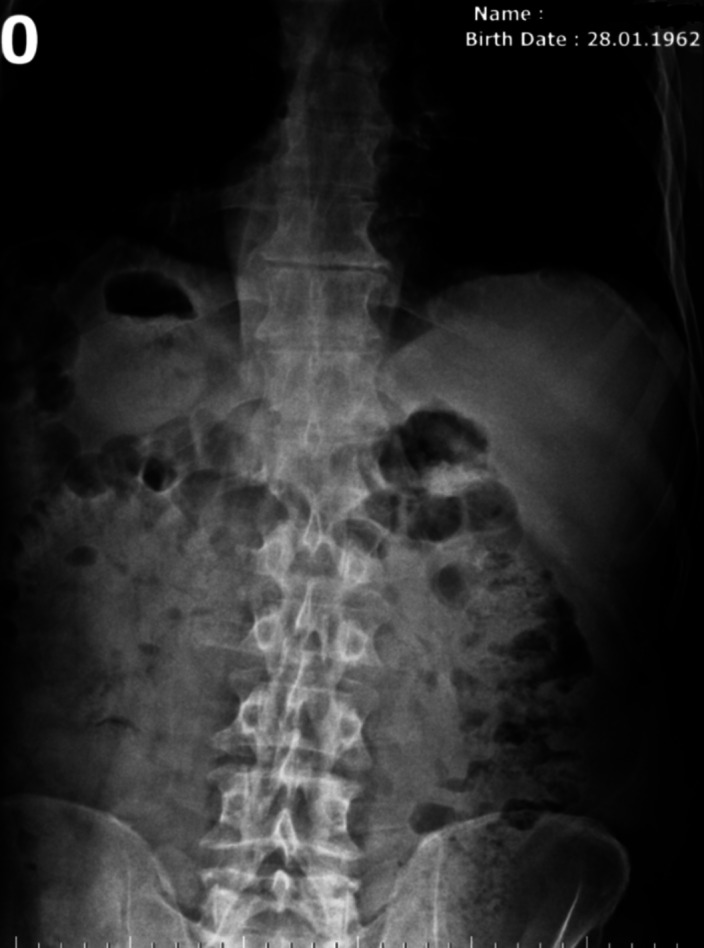

**Conclusions:**

In conclusion, the wide use of quetiapine in psychiatry requires us to be careful about such serious adverse effects. Especially in elderly patients and those with comorbid conditions, adverse effects should be closely monitored and the patient should be informed in advance of possible situations.

**Disclosure of Interest:**

None Declared

